# Health-related quality of life in patients with pediatric onset of end-stage renal disease: state of the art and recommendations for clinical practice

**DOI:** 10.1007/s00467-015-3186-3

**Published:** 2015-08-27

**Authors:** Lidwien A. Tjaden, Martha A. Grootenhuis, Marlies Noordzij, Jaap W. Groothoff

**Affiliations:** 1 Department of Pediatric Nephrology, Emma Children’s Hospital, Academic Medical Center, Amsterdam, The Netherlands; 2 Department of Medical Informatics, Academic Medical Center, Amsterdam, The Netherlands; 3 Psychosocial Department, Emma Children’s Hospital, Academic Medical Center, Amsterdam, The Netherlands

**Keywords:** End-stage renal disease, Health-related quality of life, Neurocognitive functioning, Social outcomes, Renal replacement therapy

## Abstract

Health-related quality of life (HRQoL) is increasingly recognized as a key outcome in both clinical and research settings in the pediatric population with end-stage renal disease (ESRD). This review aims to: (1) summarize the current knowledge on HRQoL and socioprofessional outcomes and (2) provide strategies for incorporation of HRQoL assessment into clinical practice. Studies report that pediatric patients with ESRD have significantly lower HRQoL scores compared with children with other chronic diseases. Patients treated by dialysis are at particularly high risk for impaired HRQoL. Furthermore, patients more often have impaired neurocognitive functioning and lower academic achievement. Important determinants of impaired HRQoL include medical factors (i.e., receiving dialysis, disabling comorbidities, cosmetic side effects, stunted growth), sociodemographic factors (i.e., female gender, non-Western background) and psychosocial factors (i.e., noneffective coping strategies). Contrary to the situation in childhood, adult survivors of pediatric ESRD report a normal mental HRQoL. Despite this subjective feeling of well-being, these patients have on average experienced significantly more difficulties in completing their education, developing intimate relationships, and securing employment. Several medical and psychosocial strategies may potentially improve HRQoL in children with ESRD. Regular assessment of HRQoL and neurocognitive functioning in order to identify areas in which therapies and interventions may be required should be part of standard clinical care.

## Introduction

Medical and surgical advances have led to dramatic changes in physical outcomes and substantial improvement in survival rates for children with end-stage renal disease (ESRD) [1, 2]. Consequently, more children with ESRD are living with the burden of long-term renal replacement therapy (RRT), a time-consuming and invasive treatment that places high demands on children and their families. Outcomes typically measured in clinical trials include survival rates and biochemical and physiological values. However, a focus on health-related quality of life (HRQoL) is needed to address the psychosocial well-being of these children and to understand the impact of the disease and its treatment on their lives.

HRQoL is therefore increasingly recognized as a key outcome in both clinical and research settings in the pediatric ESRD population [3–5].

Accurate measurement of HRQoL in children, however, has proven to be complex, and different tools have been used to this end. The purpose of our review is to give an overview of knowledge on HRQoL, neurocognitive functioning, and other social outcomes in pediatric ESRD patients (as assessed by themselves and by their caregivers) and to provide strategies for incorporation of HRQoL assessment into clinical practice. 

## Quality of life: concepts and measurement instruments

Based on the World Health Organization’s definition, QoL has been conceptualized as a multidimensional and subjective construct that reflects an individual’s subjective assessment of several domains of his or her life, including physical, social, and psychological functioning [6]. It is a broad-ranging concept affected in a complex way by the person's physical health, psychological state, level of independence, social relationships, and personal expectations. The restriction to HRQoL refers to the inclusion of dimensions that are relevant to health and are affected by ill health, such as dimensions of mobility, pain, and psychosocial well-being [7].

Several different approaches have been reported to assess HRQoL in the pediatric ESRD population, including health status measurements that evaluate one or more health domains [8–16] and qualitative methods [17, 18] (Table 1). Health status measurements can be structured as either generic or disease specific. Generic measures such as the Pediatric Quality of Life Inventory, version 4.0 ( PedsQL 4.0), the TACQOL [TNO-AZL (Netherlands Organisation for Applied Scientific Research Academic Medical Centre) Children's Quality Of Life Questionnaire], and the Child Health and Illness Profile-Adolescent Edition (CHIP-AE), are designed to assess and compare the health status of patients with different diseases and may provide valuable information for comparing outcomes between sick and healthy populations. They are generally well validated and reliable but are often not recommended for work involving evaluation of randomized controlled trials, as they lack sensitivity to detect small but clinically significant changes over time or due to treatment for specific diseases. Disease-specific measurements, like the PedsQL ESRD 3.0, are more suitable to assess the impact of a particular disease or treatment. These measures include items that are likely to be affected by the specific disease and are therefore more responsive to clinically significant changes [5].

**Table 1:** Summary of commonly used quality of life measurements in the pediatric end-stage renal disease (ESRD) population

Measure	Age range (years)	Length	Content	Range of Cronbach's α across domains Child report*	Range of Cronbach's α across domains Parent report*	Remarks
Generic measures of quality of life						
Pediatric Quality of Life Inventory (PedsQL) [19]	CR: 8–18 PR: 2–18	23 items	Physical functioning Emotional functioning Social functioning School functioning	0.68-0.88	0.75-0.90	Brief, valid, reliable, and developmentally appropriate measure. Translated into many languages Normative samples available in several countries
Child Health and Illness Profile-Adolescent Edition (CHIP-AE) [20]	CR: 10–18	107 items	Satisfaction Discomfort Resilience Risks Disorders Achievement	0.79-0.92	NA	Satisfactory psychometric properties Domain specifically relevant for the adolescent population
Child Health Questionnaire (CHQ-PF50) [21]	CR: 11–18 PR: 11–18	50 items	Physical functioning, Limitations in schoolwork and activities with friends General health Bodily pain Discomfort Limitations in family activities Emotional/time impact on the parent Impact of emotional or behavior problems on schoolwork and other daily activities Self-esteem Mental health Behavior Family cohesion Change in health	0.75-0.90	0.70-0.93	Child and parent-proxy versions Available in different languages Normative samples available in several countries
TNO-AZL Children’s Quality of Life questionnaire (TACQoL) [22]	CR: 6–11 PR: 6–15	42 items	Pain and symptoms Motor function Autonomy Cognitive and social function Positive and negative emotions	0.59-0.86	0.71-0.89	User friendly, satisfactory reliability of the parent reports
Disease-specific measures of quality of life						
Pediatric Quality of Life Inventory-End Stage Renal Disease (PedsQL ESRD module) [12]	CR: 5–18 PR: 2–18	34 items	General fatigue about my kidney disease Treatment problem Family and peer Interaction Worry Perceived physical appearance Communication	Not reported	Not reported	Includes specific ESRD or ESRD treatment-related issues Child and parent-proxy versions available
Qualitative methods (e.g., in-depth interviews)	NA	NA	NA	NA	NA	Elicits in-depth insights about the impact of the disease and treatment from the patients perspective

*CR* child report,* PR* parent report,* NA* not applicable

* Cronbach's α based on studies conducted among children with different chronic illnesses

To assess the quality of HRQoL questionnaires, psychometric properties including reliability and validity are used. Reliability refers to the degree to which an assessment tool produces stable and consistent results and is measured by Cronbach’s α. Measures with a Cronbach’s α ≥0.7 are considered sufficiently reliable [23]. Even though none of the questionnaires used in the pediatric ESRD population have specifically been validated in this population, all questionnaires do fulfil these criteria based on studies conducted in children with other chronic diseases [19–22, 24, 25] (Table 1). Finally, more qualitative methods (i.e., in-depth interviews and focus groups) have been used to elicit in-depth and detailed insights about the impact of the disease and treatment from the patient’s perspective, much of which remain unspoken during clinical encounters. These methods might expose issues that are not included in questionnaires and therefore provide a broader scope patient needs and feeling of well-being.

Measuring HRQoL in children is challenging. The relevant domains of pediatric HRQoL questionnaires differ from those in the adult population and need to be adjusted to the child’s specific developmental age. Furthermore, a common methodological criticism on published pediatric HRQoL studies is the use of a single informant, i.e., either caregiver or child. Observations of caregiver–child disagreement about HRQoL, particularly prominent in adolescent populations, have led to endorsement of multi-informant ratings of HRQoL [26–28]. Other limitations with regard to HRQoL research are related to the small sample sizes used in most studies. It is therefore difficult to generalize findings to a larger population and to undertake valid subgroup comparisons (i.e., based on medical history, social economic status, etc.). Finally, most studies use a cross-sectional design in which changes over time are not assessed. Therefore, definite information about the effect of time cannot be provided.

## HRQoL in children with ESRD

A growing number of studies have assessed the impact of RRT on the lives of children with ESRD [9, 10, 12, 13, 15, 17, 18, 29–34]. These children have to face several challenges, including frequent hospitalization, painful medical procedures, school absence, and restriction of activities. As a result, they are vulnerable to a multitude of short- and long-term academic, behavioral, and negative emotional outcomes, such as withdrawn behavior. According to several studies conducted in both Europe and the USA, pediatric ESRD patients have significantly lower HRQoL scores compared with healthy children of the same age, especially concerning physical functioning in those treated by dialysis [11–13, 29, 32, 35]. Furthermore, a study from the USA of 2500 pediatric patients from ten different physician-diagnosed disease clusters [36] found that patients with ESRD had significantly worse HRQoL scores compared to children with other chronic illnesses (including diabetes, cardiac conditions, asthma, and severe obesity) on the PedsQL 4.0 domains of physical health, psychological health, social functioning, and school functioning.

## Important determinants of HRQoL and proposed interventions

### Medical factors

As expected, the most important medical determinants associated with impaired HRQoL in the pediatric ESRD population are being on dialysis and the existence of comorbidities, such as cardiovascular diseases, underlying syndromes, urological malformations, endocrinologic disorders, and gastrointestinal diseases [8, 10, 11, 29, 37]. Due to small number of patients, most studies could not draw any conclusion about the impact of individual comorbidities. However, in particular, comorbidities that tend to represent an obstacle to social activities, such as urological malformations leading to urinary incontinence and the necessity of undergoing catheterization, may be prone to negatively affect HRQoL [37, 38].

Studies report statistically significantly higher HRQoL scores in children with a functioning renal graft, including improved physical activity, greater satisfaction, less discomfort, and better school functioning [8, 13–16, 29, 34, 39, 40]. Some studies even report similar HRQoL scores in pediatric patients with a renal transplant compared with the general population [11, 16]. However, contrary to these high generic scores, data from the disease-specific PedsQL 3.0 ESRD module reveal significant specific problems among transplanted children of all ages. These children reported a high level of concern regarding physical appearance as well as problems attributed to kidney issues (e.g., swelling, thirst, headache), and they have more frequent school disruption and difficulties with family and peer interaction [10, 13, 31]. Not surprisingly, patients who experienced a rejection episode reported the most physical discomfort [11]. The consequences of cosmetic side effects of immunosuppressive therapy, such as weight gain, gingival hypertrophy, acne, and cushingoid appearance are important but underestimated problems in patients with a functioning renal graft, particularly in the adolescent population [29, 33, 41–44] (Table 2). Proposed strategies to minimize the consequences of medication side effects and thereby improve HRQoL include managing changes in appearance by learning cosmetic application techniques and combating weight gain through physical exercise [45].

**Table 2:** Determinants of health-related quality of life (HRQoL) and proposed interventions to improve HRQoL in the pediatric end-stage renal disease (ESRD) population

Determinants	Association with HRQoL	Proposed interventions
Medical factors		
Being on dialysis (independent of dialysis modality)	Patients on dialysis reported worse HRQoL scores compared with patients with a functioning transplant. No difference was found between patients on hemodialysis and on peritoneal dialysis	The possibilities of home HD and nocturnal hospital HD should be actively explored
Cosmetic side effects of immunosuppressive therapy	Weight gain, gingiva hypertrophy, acne, and cushingoid appearance are important but underestimated problems in patients with a functioning renal transplant	Managing changes in appearance by learning cosmetic application techniques and combating weight gain through physical exercise are warranted
Stunted height	Shorter patients reported lower self-esteem and satisfaction with health. In addition, height gain and growth hormone use were associated with increases in physical and social functioning by parent-proxy report	Interventions proven to be effective in preventing growth retardation include recombinant growth hormone therapy and steroid-free immunosuppressive regimens
Anemia	Parents of anemic children reported worse HRQoL for their children and patients reported lower physical functioning	Correction of anemia by administration of erythropoietin and iron preparations may significantly improve long-term health outcomes and corresponding HRQoL.
Sociodemographic factors		
Gender	Female patients indicated they struggled more emotionally (regardless of length of time on dialysis) and had more concerns about appearance being negatively affected by their disease	Pediatric ESRD programs should direct more psychosocial resources and interventions for those particular vulnerable groups of patients and families that may benefit from greater attention
Ethnic background Parental level of education Nonintact household	Children of non-Western origin were at risk for impaired HRQoL on emotional and school functioning Low parental level of education may result in decreased HRQoL in their children Both patients and parents of nonintact households reported lower emotional HRQoL than samples from married households	
Psychosocial factors		
Coping strategies Contact with fellow patients	Obtaining knowledge, achieving a sense of normality, autonomy, and empowerment in treatment are important themes for finding ways to incorporate the disease into daily life Contact with fellow patients is important to allow children to share their feelings, coping strategies, and personal questions	Health care providers should acknowledge the specific information needs of particular adolescent patients with ESRD An interdisciplinary and coordinated plan of care that includes self-management strategies, psychosocial programs, and peer sessions facilitated by a social worker or psychologists is called for Self-management, information provision, and contact with fellow patients could possibly be delivered through innovative online initiatives

* HD* hemodialysis

In children undergoing dialysis, pronounced impairments have been reported in the domains of physical health. Surprisingly, most studies did not find significant differences in HRQoL scores between patients on hemodialysis (HD) and those on peritoneal dialysis (PD) [8, 9, 17]. It has been suggested that this lack of difference could partly be linked to the labor-intensive effort for the family associated with PD treatment, which could somewhat invalidate the advantages of more freedom and less disruption of daily life [46]. Contrary to the equality of the (low) HRQoL scores in both dialysis modalities, some studies have identified other advantages of PD over HD, such as fewer dietary restrictionsand less disruption to home- and school-based activities [47, 48]. In addition, authors of a qualitative study using in-depth interviews found that pediatric patients perceived home HD to offer greater flexibility and freedom to live a more normal life [18] compared with patients receiving long-term hospital dialysis, who indicated they felt imprisoned and were frustrated about the restrictive and unrelenting dialysis schedule. In a study conducted in the USA, home nocturnal HD was found feasible for selected adolescents, and it improved their QoL and school attendance but increased family burden [49]. Currently, pediatric home HD programs are not readily available. This is partly due to resource and logistic constraints but also because they require a substantial commitment by the training center, patient, and his or her family [50, 51]. A recent study suggests that hospital-based nocturnal HD might be another alternative, as it can offer better uremia-associated outcomes, reduces dietary and fluid restrictions, and improved QoL and school attendance [52].

Other medical factors associated with HRQoL scores include (final) height and hemoglobin level. In a Spanish study of 81 pediatric ESRD patients, the authors found that shorter patients reported lower self-esteem and lower satisfaction with health [11]. In addition, a 2-year follow-up study [53] measured the impact of short stature on HRQoL in 483 children with chronic kidney disease (CKD) and revealed that height gains and growth hormone use were associated with increases in physical and social functioning, according to parent-proxy report. Since recent data show that 43 % of patients with childhood-onset ESRD do not achieve an adult height within the normal range [54], there is certainly room for improvement with respect to reducing the extent of stunted growth. Treatment strategies shown to be effective in preventing growth retardation in patients with ESRD consist of correcting any nutritional, water, and salt deficiencies and metabolic abnormalities, especially in young infants [55]. Also, recombinant growth hormone (rhGH) therapy is safe and efficacious in children [56]. However, a recent European study showed that in several European countries, only a minority of children on dialysis with short stature actually received rhGH, in contrast to local policies [54]. Pediatric nephrologists should therefore more vigorously implement these growth-stimulating therapies in their management of children with ESRD.

Physicians should also pay sufficient attention to the correction of anemia. Parents of anemic children with CKD reported worse HRQoL scores [57], and other studies found lower physical functioning in anemic patients receiving dialysis [58] or with a functioning transplant [11]. Correcting anemia by administering erythropoietin and iron preparations may significantly improve long-term health outcomes for children with kidney disease, resulting in improved HRQoL [59, 60].

## Sociodemographic factors

Female gender is associated with impaired HRQoL in the pediatric ESRD population [9] (Table 2). Girls indicated more often that they struggled emotionally and had concerns about their appearance being negatively affected by their disease, compared with their male counterparts. Surprisingly, neither age at the time of investigation or at the start of RRT could be associated in any way with HRQoL scores in pediatric patients with ESRD [10, 32, 36].

A study conducted in children with ESRD in The Netherlands, Belgium, and part of Germany [35] found that children of non-Western origin were at risk for impaired HRQoL on emotional- and school-functioning domains. Also, low parental level of education may result in a decreased HRQoL in their children's childhood [61], and both patients and parents being part of nonintact households reported lower emotional HRQoL than those from married households [8]. Pediatric ESRD programs should therefore direct more attention in terms of psychosocial resources and interventions to particularly vulnerable families (Table 2).

### Psychosocial factors

Children with chronic illnesses use different coping strategies to deal with stressors in relation to their disease [62]. It has been suggested that by finding a way to incorporate the disease into their daily lives, these children might be able to adjust to some extent to their life-long chronic disease status. In the pediatric ESRD population, a few important themes have been identified, such as obtaining knowledge, achieving a sense of normality, and gaining autonomy and empowerment in the treatment [33]. Several studies conducted in the adolescent renal transplant population indicate that patients desire comprehensive information on technical, medical, and experimental aspects of surgery and posttransplant follow-up as well as on the effect on their physical appearance and of alcohol, drugs, and substance use on their disease [33, 34, 63, 64]. In addition, a study examining experiences and perspectives of adolescents waiting for a kidney transplant [18] identified strong apprehension about accepting a kidney from a living-related donor. The benefits of kidney transplantations were recognized, but the participants’ optimism was partly quelled by concerns about the risks to their live donor in combination with the uncertainty about transplant survival. Health care providers should acknowledge these specific informational needs, and counselling should be integrated into clinical care to address and allay fears about kidney transplantation.

The overall lower HRQoL values among children receiving dialysis may reflect their sense of lack of control, social isolation, poor self-esteem, perceived inability to gain independence, uncertainties about their health and future, and lifestyle restrictions attributable to the time-consuming and onerous dialysis regimen [65]. An integrated clinic with ancillary services that address sufficient school and psychosocial support are therefore recommended and might even result in better clinical outcomes [66]. This also has important implications for transitional care, as the adult model of care may not address cognitive, emotional, and social needs of young people, and transition is often poorly coordinated. Furthermore, to enhance empowerment and autonomy, we suggest shared decision-making processes in which patients are actively involved in their own treatment decisions [67].

Studies also reveal the importance of social support in terms of contact with fellow patients, as this would encourage children to share their feelings, coping strategies, and personal questions [17, 18]. Based on these findings, a community-based young adult transplant service has been established in Oxford, UK. This clinic involves a peer session facilitated by a social worker and informal social activities, while the hospital team provides an outreach medical service. Surveys conducted among participants showed that this provided a better environment for peer interaction and support, which resulted in a higher clinical attendance rate, greater patient satisfaction and engagement, and reduced episodes of late rejection [68]. Furthermore, studies in adolescents with other chronic diseases demonstrated the potential value of Internet-based programs and mobile phone communication [69–71]. These results indicate that contact with fellow patients, but also a greater sense of self-management and access to information, could possibly be delivered through innovative online initiatives.

Moreover, given current economic constraints and scarcity of psychosocial services, it is particularly important to allocate resources in a cost-effective manner. Consistent and rigorous monitoring of HRQoL in children with ESRD might reveal specific problems at an early phase, thereby preventing further deterioration. In recent decades, there has been a growing interest in patient-reported outcomes (PROs) as a tool for pediatricians to use to discuss psychosocial issues during medical consultation [72]. When pediatric psychologists and pediatricians work together, PROs can be used to closely monitor children with chronic illnesses in a multidisciplinary context, and referral to psychosocial interventions can be better facilitated [73].

## Neurocognitive status, educational attainment, and social activities

Neurocognitive dysfunction is a well-recognized complication of pediatric ESRD. Many studies found evidence of several neuropsychological deficits, including low scores for intelligence quotient (IQ), academic achievement, memory, and executive functioning [74–76]. In a large North American Study [Cronic Kidney Disease in Children (CKiD) of 386 children with CKD 2–4 (mean GFR 41), the overall neurocognitive functioning was comparable with the general population, but 21–40 % of participants scored at least one standard deviation (SD) below the mean for IQ, academic achievement, attention regulation, or executive functioning [77]. The risk of developmental delay may be greatest for infants and young children on dialysis therapy because infancy is a period of rapid neurodevelopment, and approximately one half of brain growth takes place during the first years of life [78–81]. Therefore, early intervention programs designed to improve specific developmental areas, such as language abilities and motor function, should be sought. The neurodevelopmental status of older children and adolescents on dialysis therapy has been characterized by abnormal performance on tasks of verbal abstract ability, visual perceptual reasoning, memory, and visual motor skills compared with healthy children and transplant recipients [82]. It is therefore recommended that patients on dialysis therapy have age-appropriate cognitive and achievement testing conducted on a regular basis, especially if problems with school achievement exist.

Not surprisingly, ESRD and its treatment often decrease participation in peer and school-based activities. In a Turkish cohort of 211 children with ESRD, 50 % of patients treated with dialysis and 37.9 % of transplanted patients had left school without grading because of their illness [14]. This is in line with reports from the USA, where only 53 % of patients receiving HD were able to successfully achieve full-time school attendance [83]. School attendance rates were higher among transplanted patients; however, even in this group, the impact of ESRD on academic functioning remained of particular concern for both patients and parents [9]. In addition, prolonged absence from school due to treatment-related issues can lead to a detrimental effect on sustaining peer relationships, self-esteem, and academic achievement, which negatively influences QoL [12, 15, 40, 84]. Therefore, regular school attendance should be a primary outcome measure of the overall success of RRT in children. Especially among school-aged children treated by dialysis, there is a need for strategies to optimize education opportunities. Dialysis units could have on-site educators to complement school-based teaching. There could be a close liaison with schools to offer appropriate support in reducing school absenteeism to a minimum. As noted earlier, attention could also be paid to identifying neurocognitive or behavioral abnormalities that may have a significant negative impact on the likelihood of success in the classroom. A psychologist-directed assessment can be particularly helpful as a means of detecting specific learning difficulties and targeting particular educational strategies [85].

## Child versus parent-proxy scores

While the child’s self-report should be considered the standard for measuring perceived HRQoL, there are circumstances, such as young age of the patient (<5 years old) and cognitive impairment, in which a child is unable to complete a HRQoL instrument. In those cases, parent-proxy reports are mandatory. Scores from parents are not only relevant as a substitute for the child’s HRQoL perception, but they also provide a more complete and comprehensive picture of the impact of the disease on the family. Second, they are important because it is typically the parents’ perception of their child’s well-being that influences health care use [86]. Therefore, using instruments that measure both parent and child perspectives is recommended. However, studies reveal a statistically and clinically significant discordance between parent and child HRQoL scores, with patients reporting higher scores than their caregivers [10, 87, 88]. Disagreement between parents’ and children's reports of HRQoL is, in itself, unlikely to indicate that one of them is wrong or right, but rather is a consequence of different beliefs about the child's health and well-being. Due to their own grief about unfulfilled expectations for their child and concerns regarding the (long-term) impact of ESRD, parents may have a more negative view than their children [89]. A recent meta-analysis regarding discrepancies between child and parent-proxy HRQoL scores suggested that the differences in scores also depends on the HRQoL domain studied [86]. Parents tended to be more in agreement with their children on objective physical domains but less so in terms of emotional and social functioning and symptoms such as pain, fatigue, and gastrointestinal complaints. Limited knowledge due to respect for the child’s privacy and autonomy may also be a factor accounting for the observed differences. In line with this hypothesis, a study in the UK among 428 pediatric patients with CKD and their parents [53] showed that concordance between child and parent perceptions of HRQoL was age dependent, with a lower correlation between self- and parent-proxy scores among adolescent patients compared with younger patients with ESRD. Obviously, the parents’ own well-being may affect their perception of their child’s HRQoL and symptoms. A poorer QoL, difficulties in managing the child’s care, and higher levels of anxiety and maladaptive behavior have been identified in parents of children with ESRD [44, 90], which could result in a more negative view regarding their child’s well-being. Therefore, sufficient support for families should also be incorporated into standard clinical care. This may prevent parent overprotectiveness and may, as such, indirectly lead to better medical outcomes and HRQoL in the child [91].

## Children reaching adulthood

Few studies have reported on the effect of RRT in childhood on HRQoL and social functioning in adulthood. According to two studies [92, 93], adult patients with a functioning kidney transplant achieve normal scores on self-assessment of psychosocial and physical health. In a Dutch cohort study on the long-term effects of renal insufficiency in children (LERIC) we found that after 20–40 years of RRT [92], the proportion of impaired HRQoL in transplanted patients was only higher for the domain of general health perception when compared with the aged-matched general population. Mental health of transplanted patients was even significantly better than in the general population. Equally good scores were found in an Italian follow-up study on transplanted patients aged 18–34 years who were transplanted at a median age of 15 years [93]. Dialysis patients from the LERIC study more often had impaired scores compared with the general population for physical domains, but scores on mental domains were similar or even better. Also, in young adults (median age 19.7, range 16.3–23.4 years) in the UK who survived severe CKD in infancy, significantly lower scores on physical functioning but similar scores on emotional well-being compared with the age-matched general population were found [37]. These high scores on emotional domains are in contrast with scores from patients with adult-onset ESRD treated by dialysis who reported substantially lower scores in the domains related to mental HRQoL [94]. It has been suggested that this difference could be directly related to a nearly life-long chronic disease status: patients with a chronic disease since childhood may have grown up with a lower perception of life than those who acquired a chronic disease in adulthood. Consequently, expectations regarding life of patients in the former group are probably better met, despite physical disabilities.

In terms of social and professional outcomes, several studies have shown that young adult survivors of childhood ESRD struggle to secure gainful employment, complete their education, experience intimate relationships, and live independently [95–102]. A French study assessing 374 adult (aged 24–36) survivors of pediatric transplantation found that professional occupation was similar to the French general population but the unemployment rate was higher: 18.5 % vs. 10.4 %; *P* < 0.01. This rate is comparable with findings in Dutch adults with ESRD since childhood (19.4 % vs. 11.1 % in the healthy population;* P* < 0.01 [92]) and with findings from a German study in 120 middle-aged survivors of pediatric ESRD (14.0 % vs. 9.0 % in the healthy population;* P* < 0.01) [101]. All studies found relatively lower educational attainment in patients compared with the general population, which partly explains the disadvantage in gaining employment. The LERIC study also found that the low income rate (below national average) was significantly higher in patients than in the general population. Remarkably, the study conducted in the UK among adolescent survivors of severe CKD in infancy found an unemployment rate comparable with the general population (17 % vs. 16.2 %) [37]. The discrepancy with the French and Dutch data might be partly explained by the younger age at time of investigation in our cohort. As a consequence, the majority of patients were still studying, and only 24 % had a paid job.

## Conclusion and recommendations

ESRD and its treatment have a severe impact on the HRQoL of children, as it affects both physical and emotional functioning and interferes with normal daily life. In particular, patients on dialysis are at high risk for impaired HRQoL. Regular assessment of HRQoL, including implementation of patient-reported outcomes, should therefore become part of the standard care in order to identify specific areas for interventions. Potential effective medical and psychosocial strategies are known to improve HRQoL, but more attention must be paid to implementing these strategies. Medically, more effort must be put into implementing treatment regimens that reduce cosmetic side effects and the poor growth rate in children with ESRD. Where the possibility of a successful renal transplant is unlikely, a home HD program that can reduce dietary and fluid restriction and improve freedom and autonomy should be actively explored. Several psychological interventions and initiatives exist that may stimulate such children’s ability to cope with ESRD and its treatment by providing capacity and encouraging confidence to manage their own health, participate in social activities, progress in their studies, and remain vigilant in dialysis and posttransplant treatment responsibilities. All studies advocate a multidisciplinary patient-centered approach that promotes shared decision making, control, and self-efficacy in treatment management and educational and vocational opportunities.

Furthermore, several neurocognitive problems, including deficits in IQ, academic achievement, and executive functioning, have been reported in the pediatric ESRD population. Especially for children on dialysis, it is recommended to conduct age-appropriate cognitive and achievement testing on a regular basis. Dialysis units should have on-site educators to complement school-based teaching.

In the long term, adult survivors of pediatric ESRD have impaired physical but encouragingly good mental HRQoL. However, they do experience more difficulties integrating into the working population and building a social and family life.

## Research agenda

Large multicenter and longitudinal studies are warranted to ensure adequate sample size and statistical power to better elucidate changes over time in HRQoL outcomes, to investigate which patient factors (i.e., medical, sociodemographic, and psychosocial) may be related to such change, and to further assess which interventional targets would be most suitable. The implementation of patient-reported outcomes might be a feasible platform to closely monitor HRQoL outcomes over time. Studies should use both generic and disease-specific questionnaires and obtain information from both patients and parents in order to generate the most comprehensive outcomes. Furthermore, a utility-based approach has recently been used to assess QoL among (pediatric) ESRD patients. This construct assesses the relative desirability of health states and forms the basis of the quality-adjusted life year (QALY) analysis, commonly used by health economists in decision- and cost-effectiveness studies. This approach is a promising addition to the existing health status measurements. However, since these questionnaires have only been used in a few studies among pediatric ESRD patients, further research is required to assess their validity and reliability in this specific population.

Future research should focus not only on evaluating the effect of the proposed interventions longitudinally on HRQoL but also on relevant medical outcomes, such as adherence, biochemical control, and graft survival, thererby acknowledging the difficulties of such studies with so many co-variables.

## Key summary points

1. ESRD in children affects physical and emotional functioning, with an adverse impact on HRQoL and neurocognitive functioning, especially for patients receiving dialysis.
2. Determinants of impaired HRQoL include medical, sociodemographic, and psychosocial factors.
3. Rigorous monitoring of HRQoL and cognitive testing might reveal specific problems at an early stage, facilitating appropriate interventions.
4. Implementation of treatment regimens that reduce cosmetic side effects and poor growth should be pursued.
5. Psychosocial interventions are warranted to stimulate coping strategies to give children the capacity and confidence to manage, and gradually take responsibility for, their own health, as well as progressing in their studies and participating in the wider community.

## Questions (Answers are provided following the reference list)

1. According to the current literature, the overall HRQoL of patients on PD compared with patients on HD is:
  - Less impaired
  - Equally impaired
  - More impaired
  - No sufficient evidence
2. Which medical factor has** not** been associated with impaired HRQoL?
  - Final height
  - Side effects of immunosuppressive regimen
  - Level of serum hemoglobin
  - Level of serum urea
3. Compared with parent-proxy HRQoL scores, patients report:
  - Higher HRQoL scores
  - Lower HRQoL scores
  - Comparable HRQoL scores
  - Scores are never obtained from both patients and caregivers
4. Which statement is true regarding sufficient support of adolescent patients?
  - Adolescent patients should be consciously checked and overheard to prevent them from not following medical recommendations
  - Adolescent patients should be encouraged to manage their own health and to take responsibility in their treatment
  - Adolescent patients often do not want to share their experiences and coping strategies with other patients
  - Adolescent patients should not be informed about risk-taking behaviors, as this could make such behaviors more likely to be attempted
5. The risk for developmental delay regarding neurocognitive functioning is the highest for patients with ESRD onset at age:
  - <1 year
  - 1–2 years
  - 2–5 years
  - >6 years
6. Adult survivors of pediatric ESRD report:
  - High scores on both emotional and physical HRQoL domains
  - Low scores on both emotional and physical HRQoL domains
  - High scores on emotional domains but low scores on physical HRQoL domains
  - Low scores on emotional domains but high scores on physical HRQoL domains
